# The MTRNR2L gene family and age-related macular degeneration

**DOI:** 10.3389/fimmu.2026.1925795

**Published:** 2026-09-07

**Authors:** Adam Moosa, Joshua J. Wang, Margaret M. DeAngelis, Sarah X. Zhang

**Affiliations:** 1 Department of Ophthalmology and Ross Eye Institute, Jacobs School of Medicine and Biomedical Sciences, University at Buffalo, State University of New York, Buffalo, NY, United States; 2 Neuroscience Program, Jacobs School of Medicine and Biomedical Sciences, University at Buffalo, State University of New York, Buffalo, NY, United States; 3 Department of Biochemistry, Jacobs School of Medicine and Biomedical Sciences, University at Buffalo, State University of New York, Buffalo, NY, United States; 4 Genetic, Genomic and Bioinformatics Program, Jacobs School of Medicine and Biomedical Sciences, University at Buffalo, State University of New York, Buffalo, NY, United States

**Keywords:** AMD (age-related macular degeneration), cytoprotection, humanin, mitochondrial derived peptides, MTRNR2L, oxidative stress, retina pigment epithelial cells

## Abstract

Age-related macular degeneration (AMD) is the leading cause of blindness in aging adults, yet no FDA-approved therapies exist for early and intermediate disease. Multiple interconnected pathways, including mitochondrial dysfunction, oxidative stress, and inflammation, contribute to retinal pigment epithelium (RPE) degeneration in AMD, highlighting the need for developing new cytoprotective strategies. The MTRNR2L gene family, comprising nuclear paralogs of mitochondrial-derived peptide humanin, has emerged as a potential regulator of these pathways. Humanin has demonstrated anti-apoptotic, anti-inflammatory, and mitochondrial-stabilizing effects across multiple cell types, including the RPE. Recent transcriptomic and single-cell RNA-sequencing (scRNA-seq) analyses indicate that MTRNR2L genes are upregulated in a variety of human diseases associated with cellular stress response, inflammation, angiogenesis, and neurodegeneration. These findings suggest that MTRNR2L genes participate in coordinated stress-adaptive programs and may mirror humanin’s cytoprotective and immunomodulating actions. This review summarizes recent research advances and highlights key gaps for understanding the roles of the MTRNR2L family in RPE protection and AMD progression.

## Introduction

Age-Related Macular Degeneration (AMD) is the leading cause of blindness in individuals over the age of 65 (1, 2). With the drastic increase in life expectancy and the rapid growth of the aging population, it is projected that over 288 million people will be affected by AMD by 2040 worldwide (1). Currently, no effective FDA treatments are available for early or intermediate AMD, highlighting an unmet need to develop novel therapeutic approaches to halt the progression of this blinding disease (3, 4). Among the potential strategies, preserving the retinal pigment epithelium (RPE) is particularly important because these cells carry out essential functions for maintaining a healthy retina and hence, vision. These functions include: 1) phagocytosing the shed photoreceptor outer segments as a garbage disposal, 2) recycling all-trans retinal to regenerate 11-cis retinal in the visual cycle, 3) secreting growth factors to maintain choroidal vascular health and producing trophic factors to nurture photoreceptors, 4) forming the outer blood-retina barrier (BRB) to sustain a homeostatic microenvironment for the neuroretina, and 5) providing metabolic support to photoreceptors. Dysfunction of the RPE can cause toxic byproduct accumulation, leading to enhanced endoplasmic reticulum (ER) stress, oxidative stress, mitochondrial damage, and chronic inflammation, ultimately resulting in photoreceptor death and central vision loss in AMD (5–7).

Humanin (HN) is a small, secretory polypeptide encoded by the mitochondrial gene MTRNR2. Humanin has demonstrated potent cytoprotective effects in experimental models of neurodegenerative disorders, including Alzheimer’s disease and AMD, and diabetes (8–10). These protective effects are attributed primarily to its anti-oxidative and anti-apoptotic activities, as well as its ability to increase mitochondrial metabolism and enhance cellular stress resistance (11). A recent study showed that a synthetic analog of humanin, Humanin-G, may possess even more potent protective effects by suppressing inflammation associated with AMD pathogenesis (10). These encouraging findings have prompted researchers to explore the genetic implications of MTRNR2 and other related genes in AMD. Recent work by Shwani et al. revealed that the expression of a nuclear-encoded humanin isoform gene (MTRNR2L1) is significantly increased in the macula of deeply phenotyped human donor eyes with intermediate or neovascular AMD, while the expression of the mitochondrial MTRNR2 remains unchanged (12). Differential expressions of MTRNR2L genes and proteins have also been identified in other human neurodegenerative diseases and inflammatory diseases, such as ulcerative colitis (13), Alzheimer’s disease (14), tauopathies (15), and multiple sclerosis (16). In Down syndrome–associated Alzheimer-like dementia, MTRNR2L12 expression is significantly higher in patients with good or acceptable cognitive status compared to those with severe cognitive disability; this correlation suggests a potential role of MTRNR2L12 in maintaining cognitive function during dementia progression (17). In this review, we summarize recent advances and existing knowledge gaps in MTRNR2L research, emphasizing the significance and potential implications of the MTRNR2L gene family in AMD and other forms of retinal degeneration, inflammatory/neuroinflammatory disorders, and systemic human diseases.

## The MTRNR2/MTRNR2L family: discovery and function

Humanin is the first mitochondria-derived peptide (MDP) discovered in 2001 through a functional screening study aimed at identifying protective factors against Alzheimer’s neuronal cell death (8). It was named with the hope of restoring the ‘humanity’ of patients with Alzheimer’s disease (8, 18). The peptide is encoded by a 75-bp open reading frame (ORF) within the mitochondrial 16S rRNA gene (MTRNR2) located in the genome. Depending on the translational site, humanin can be produced either as a shorter 21-amino-acid peptide if translated in the mitochondria or a longer 24-amino-acid peptide if translated in the cytoplasm, and both forms exhibit cytoprotective functions (19).

Over the past two decades, humanin has generated substantial scientific interest due to its potent neuroprotective effects in Alzheimer’s disease models (2, 8). Studies have revealed multiple mechanisms underlying humanin’s cytoprotective effects, including anti-oxidative, anti-apoptotic, and mitochondrial-stabilizing activities (2, 8–11). Mechanically, humanin activates the PI3K/AKT and JAK/STAT3 pathways to enhance cellular survival and inhibit apoptosis by suppressing Bax translocation to mitochondria (20, 21). Additionally, secreted humanin binds to formyl-peptide receptor-1-like 1 (FPRL1) on the cell surface and activates heterotrimeric G proteins, eliciting neuroprotective effects (18).

Recent *in silico* analyses of the mitochondrial genome have identified six small humanin-like peptides (SHLP1-6) (11). Like humanin, these SHLPs are encoded within the mitochondrial 16S rRNA region and possess similar cytoprotective properties (11, 22). These endogenous cytoprotective mitochondrial peptides, such as SHLP2, likely play an important role in maintaining mitochondrial homeostasis and enhancing cellular resistance to stress-induced injury, thereby protecting retinal cells during AMD pathogenesis (23).

The nuclear paralogs of MTRNR2, namely the MTRNR2L gene family (L1-L13), were identified in 2009 through bioinformatic analyses of the human nuclear genome. These analyses revealed 13 loci predicted to encode full-length functional humanin-like peptides out of a total of 28 nuclear MTRNR2L sequences. The remaining 15 sequences contain premature stop codons or lack consensus translation start sites (24). Historically, the MTRNR2L gene family was categorized as nuclear mitochondrial DNA sequences (NUMTs) and considered pseudogenes. This study demonstrated that at least 10 MTRNR2L genes are transcriptionally active in various human tissues, with expression particularly high in the testis, heart, skeletal muscle, brain, and kidney, and relatively low in the liver, thyroid gland, and bone marrow (24). Notably, mitochondrial MTRNR2 expression is 100-250,000-fold higher than nuclear isoform expression across all tested tissues (24). Cross-species analyses identified 14 full-length homologues in chimpanzees and 20 in rhesus monkeys, demonstrating conservation within primate lineages (24). Functional studies revealed key amino acids, e.g., proline at position 19, influencing peptide secretion and threonine at position 13, responsible for cell surface receptor binding (24).

Functional testing using synthesized humanin 5 (HN5) peptide variants, encoded by MTRNR2L5, demonstrates cytoprotective activity against staurosporine-induced apoptosis in endothelial cells, supporting the possibility that some MTRNR2L transcripts may encode biologically active peptides (24). However, direct evidence of endogenous humanin-like peptides is currently lacking, leaving open the possibility that some isoforms may function as noncoding RNAs with roles in stress regulation (25, 26). Nevertheless, recent transcriptomic and single-cell RNA sequencing (scRNA-seq) studies have shown differential expression of multiple MTRNR2L genes, including MTRNR2L1, in the RPE, retina, and neural tissues across human diseases involving aging, mitochondrial dysfunction, oxidative stress, protein misfolding, and endoplasmic reticulum stress (12–15, 27, 28). These findings establish a reproducible transcriptional association between MTRNR2L genes and cellular stress response, mitochondrial function, and redox regulation, independent of their precise molecular mechanisms of action.

The MTRNR2L paralogs encode predicted peptides that differ from humanin, and from one another, by only a few amino acids. Antibodies and ELISA platforms used in the literature, whether raised against humanin or marketed as paralog-specific, have not, to our knowledge, been validated to distinguish a given MTRNR2L peptide from humanin or from other paralogs, and no study confirms the identity of the detected species by mass spectrometry. Reported “humanin-like” peptide signals should therefore be read as aggregate humanin-family immunoreactivity rather than the product of any single locus. A related ambiguity applies to short-read transcript data, where reads can misassign across these near-identical loci. Because this limitation is shared across the literature, we flag it here and refer to it below.

## The MTRNR2L family: implications in human disease development

Recent genetic studies using advanced transcriptomic and scRNA-seq approaches provide multiple lines of evidence supporting a role of the MTRNR2L family in the development and pathogenesis of several human diseases, including AMD, mitochondrial encephalomyopathy, lactic acidosis, and stroke-like episodes (MELAS), pediatric glioblastoma, ulcerative colitis (UC), Alzheimer’s disease, tauopathies, and multiple sclerosis (12–16, 27, 28). **Table 1** summarizes major findings from published studies on the MTRNR2L family, highlighting their associations with human diseases, implicated cellular processes and signaling pathways, and predicted biological functions.

**Table 1:** Implications of the MTRNR2L gene family in human diseases.

Gene	Disease / Model & tissue	Cell types (if specified)	Expression direction	Modality	Functional pathways	Evidence type	Ref.
MTRNR2L1	AMD human donor eyes macular RPE/choroid neural retina	—	↑	Bulk RNA-seq	Stress response, angiogenesis, RPE–retinal coordination	Experimental	(12)
MTRNR2L1	AMD human donor eyes macular RPE/choroid	CECs, melanocytes, Schwann cells, smooth muscle cells	↑	Single-cell RNA-seq	Stress response, melanin production, oxidative stress, angiogenesis	Experimental	(30)
MTRNR2L1	MELAS human donor eyes choroid	Fibroblasts, ECs, T cells	↑	Single-cell multi-omics	Mitochondrial stress response, cell survival	Experimental	(27)
MTRNR2L1	Pediatric glioblastoma	—	Hub (expression not reported)	Regulatorynetwork inference	Central hub gene in stress response networks	Computational (Predicted)	(28)
MTRNR2L12	AMD human donor eyes macular RPE/choroid	CECs, smooth muscle cells	↑	Single-cell RNA-seq	Angiogenesis; mitochondrial/ inflammatory response	Experimental	(30)
MTRNR2L12	Down syndrome + AD-like dementia (blood)	Peripheral leukocytes	↑ (DS-AD vs DS-non-AD)	Microarray qPCR	blood biomarker; stress resilience	Experimental	(17)
MTRNR2L12	Ulcerative colitis (remission vs relapse)	Colonic mucosa	↑ in remission	RNA-seq cohorts + modeling	Anti-apoptotic / stress response	Experimental	(13)
MTRNR2L1/8	Pediatric IBD & eosinophilic GI disorders (PBMCs	Immune cells (B, CD4/CD8 T, DC, NK)	↑	Single-cell RNA-seq	Cellular-stress / active-inflammation marker	Experimental	(39)
MTRNR2L3 / L6 / L8 / L12	Ulcerative colitis (remission / relapse)	Colonic mucosa	↑ in remission	RNA-seq + survival modeling	Anti-apoptotic / stress-resilience correlating with longer remission	Experimental	(13)
MTRNR2L8	Pediatric glioblastoma (IDH-WT)	—	Hub gene (expression not reported)	Single-cell network analysis	Mitochondrial-stress adaptation and cellular plasticity	Computational (predicted)	(31)
MTRNR2L1 / L10	Experimental epilepsy (rat cortex)	—	↓ in epilepsy (protective when restored)	Bioinformatics + *in vivo* validation	Suppress NLRP3 inflammasome, anti-apoptosis, preserve BBB	Experimental	(36)
MTRNR2L3	MS-SCON vs NMOSD/RRMS (serum)	—	↑ in MS-SCON	Serum ELISA	Biomarker; neuronal stress/survival	Experimental	(16)
MTRNR2L8 / L12	Alzheimer’s disease (prefrontal cortex)	Excitatory neurons, astrocytes,	↑ in AD pathology	Single-nucleus RNA-seq	Stress-response upregulation; shared late-stage induction	Experimental	(14)
MTRNR2L1 / L2 / L3 / L8 / L10 / L12 / L13	Tauopathy (MAPT-mutant cerebral organoids)	Pyramidal neurons, astrocytes (organoids)	↑ in heterozygous; ↓ in homozygous mutant	Single-cell RNA-seq	Anti-apoptotic stress response; dose-dependent reversal	Experimental	(15)

AMD, age-related macular degeneration; DS-AD, Down syndrome with Alzheimer-like dementia; CEC, choroidal endothelial cell; EC, endothelial cells; NMOSD, neuromyelitis optica spectrum disorder; RRMS, relapsing-remitting multiple sclerosis; MS-SCON, multiple sclerosis with predominant spinal cord and optic nerve involvement.

↑ means increased expression of gene, ↓ means decreased expression.

## MTRNR2L1

**Figure 1:**
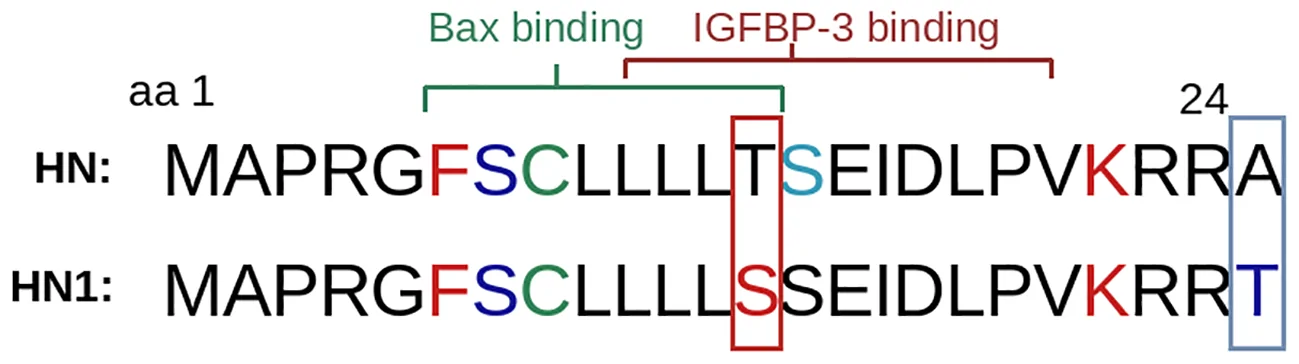
humanin 1 (HN1) peptide. Key residues for humanin (HN), including BAX binding (amino acid residue 8), IGFBP3 binding (amino acid residues 6–21), and self-dimerization (amino acid residue 7), and HN1 with amino acid differences highlighted at positions 13 and 24. Figure was inspired by (26).

MTRNR2L1, located on chromosome 17 (17p11.2), is one of the most studied members of the MTRNR2L gene family and demonstrates high similarities in tissue expression patterns with MTRNR2 (24). Structurally, MTRNR2L1/humanin 1 shares remarkable homology with mitochondrial-derived humanin, differing by only two amino acids (**Figure 1**). As a nuclear-encoded paralog, however, it exhibits distinct regulatory patterns and is consistently upregulated across diverse tissues and disease contexts, a pattern interpreted as a stress-associated transcriptional response (26). A recent transcriptomic study of duodenal samples from severely obese subjects showed that MTRNR2L1 mRNA is significantly upregulated in the intestines of insulin-resistant individuals compared to insulin-sensitive individuals (29). Although the exact role of MTRNR2L1 in intestinal physiology remains unclear, this strong correlation suggests that it may function as a regulator of metabolic stress adaptation and serve as a potential biomarker of insulin-resistant obesity.

The significance of MTRNR2L1 in AMD has been exemplified by several recent investigations. In a bulk RNA-seq study, gene expression in the macular RPE/choroid and neural retina from well-characterized post-mortem human donor eyes across AMD stages were profiled and compared with age-matched normal controls. MTRNR2L1 was the only gene significantly upregulated in both macular RPE/choroid and neural retina in intermediate AMD compared to age-matched controls, and its expression level was among the most significantly increased, compared to known stress-response markers (12). This suggests that MTRNR2L1 may represent a coordinated mitochondrial and/or oxidative stress response element activated across the macula in AMD. Moreover, MTRNR2L1 expression increased further in neovascular AMD, suggesting its potential use as a biomarker of escalated retinal stress during disease progression of AMD (12).

A recent scRNA-seq study further supports the activation of MTRNR2L1 in AMD. In human macular tissue, MTRNR2L1 is significantly elevated in several cell types in intermediate AMD, including choroidal endothelial cells, melanocytes, Schwann cells, and smooth muscle cells (**Table 2**) (30). The magnitude and breadth of this induction further highlight MTRNR2L1 as a widely activated stress-responsive transcript within the macular microenvironment, suggesting a potential role of this gene in cellular adaptation to mitochondrial and oxidative stress in AMD.

**Table 2:** MTRNR2L1/L12 expression in macular cells in intermediate AMD compared to normal.

Gene name	Cell type	p_val	Avg_logFC	pct.1	pct.2	p_val_adj
MTRNR2L1	Choroidal endothelial cells	1.98E-17	0.764	0.571	0.057	3.63E-13
MTRNR2L12	Choroidal endothelial cells	3.65E-07	0.278	0.983	1.000	6.68E-03
MTRNR2L1	Melanocytes	8.86E-14	0.979	0.400	0.027	1.62E-09
MTRNR2L1	Schwann cells	8.83E-10	1.422	0.694	0.030	1.62E-05
MTRNR2L1	Smooth muscle cells	1.18E-10	1.597	0.772	0.125	2.15E-06
MTRNR2L12	Smooth muscle cells	3.00E-07	0.746	1.000	1.000	5.48E-03

Results were generated by analyses of published data from (30).

The significance of MTRNR2L1 upregulation extends beyond AMD pathophysiology. In MELAS, MTRNR2L1 is significantly upregulated in multiple choroidal cell types – fibroblasts, endothelial cells, and T cells – independent of mitochondrial heteroplasmy levels (27). This finding indicates that MTRNR2L1 induction is not contingent on heteroplasmy burden, and is consistent with, though does not establish, a generalized transcriptional response to mitochondrial stress. In ulcerative colitis and glioblastoma, MTRNR2L family members, including MTRNR2L1, have been associated with anti-apoptotic as well as mitochondrial-protective pathways, expanding their implication beyond neurodegenerative contexts (13, 28). Across multiple diseases, MTRNR2L1 expression tends to be upregulated either during disease remission or under cellular stress, reinforcing a pattern consistent with, but not demonstrative of, a conserved stress-associated transcriptional program during metabolic, bioenergetic, or inflammatory challenges (13, 27).

In pediatric glioblastoma, MTRNR2L1 and MTRNR2L2 were identified as high-centrality nodes within the inferred glioblastoma–immune regulatory network, integrating signals from transcription factors governing glioblastoma stemness and cell fate transitions, including GATA2, OLIG1/2, and PDGFRA (28). Although its precise function remains unelucidated, the high sequence homology to humanin and its position within the stress-response networks suggest that MTRNR2L1 possesses potential neuroprotective and anti-apoptotic roles (28). Deep learning network analysis of glioblastoma single-cell transcriptomes also identified MTRNR2L1 as a feature associated with cellular plasticity in an unsupervised deep-learning analysis (31). In a transcriptomic study of resected colorectal liver metastases, MTRNR2L1 was the only gene differentially expressed at a false discovery rate below 0.1 in adjacent liver parenchyma, with higher expression in good survivors (those free of recurrence beyond 60 months), compared with poor survivors, who died of recurrent disease within 30 months (32). The authors note that the parenchymal analysis carried relatively high false discovery rates and was less reliable than the paired tumor analysis, and MTRNR2L1 was not carried forward into protein-level validation; therefore, it is consistent with, but does not necessarily establish, a tissue-protective response in the metastatic niche. Conversely, in a recent study of colorectal cancer, elevated MTRNR2L1 expression in tumor cells was associated with chemotherapy resistance, with higher levels observed in non-responders compared to responders or treatment-naïve patients (33).

Cumulative evidence suggests a multifaceted role of MTRNR2L1 in immune regulation across diverse biological contexts. In circulating monocytes, BCG vaccination induces upregulation of MTRNR2L1 as part of a coordinated immune module that enhances trained immunity, a form of innate immune memory that confers non-specific protection against heterologous pathogens (34). Notably, genetic polymorphisms in MTRNR2L1 significantly influence the magnitude of trained immune responses, with associations observed for TNF-α and IL-6 cytokine production both *in vitro* and *ex vivo* following pathogenic challenge (34). Furthermore, functional validation showed that recombinant humanin induces trained immunity in primary monocytes. Notably, this validation used recombinant humanin rather than an MTRNR2L1-encoded peptide; given their near-identical sequences, the experiment cannot attribute the effect to MTRNR2L1 specifically (34). Beyond innate immunity, MTRNR2L1 has been examined in relation to tumor immune composition. A pan-cancer analysis of TCGA data found MTRNR2L1 to be elevated in glioblastoma and associated with poorer glioma survival, with deconvolution-based analysis of the glioblastoma cohort showing a significant positive correlation with memory B cell infiltration (R = 0.197, P = 0.010) but not with CD8^+^ T cell or macrophage cell populations (35), a single preliminary analysis that cannot distinguish expression by tumor cells from expression by the infiltrating immune populations themselves. Taken together, these findings across circulating monocytes and the tumor microenvironment suggest that MTRNR2L1 may participate in immune-associated transcriptional programs in more than one cellular context, though its role in each remains to be established directly (34, 35).

Beyond these correlative findings, recent work provides the first *in vivo* functional evidence of a protective role for MTRNR2L1. In a rat model of status epilepticus, integrated bioinformatics and experimental validation identified the MTRNR2L1/MTRNR2L10 axis as a candidate regulator of cortical injury, with cortical expression of both genes falling to less than 30% of control levels in epileptic animals (36). Overexpression of MTRNR2L1 or MTRNR2L10 lowered seizure severity, reduced neurological deficit scores, reduced NLRP3 inflammasome-related marker expression, mitigated the loss of blood-brain barrier tight-junction proteins (ZO-1, occludin, and claudin-5), and decreased cortical neuronal apoptosis and oxidative stress. Whereas knockdown of either MTRNR2L1 or MTRNR2L10 made these outcomes worse (36). Co-immunoprecipitation further suggested that the two molecules associate within the same protein complex, though direct physical binding was not established (36). These findings suggest causal support for the hypothesis that MTRNR2L1 acts as a cytoprotective, anti-apoptotic, and anti-inflammatory stress-response element, consistent with its repeated upregulation across disease states as noted throughout this review.

## MTRNR2L12

Like MTRNR2L1, MTRNR2L12 has emerged as an important gene associated with cellular stress resistance and disease outcomes across multiple biological systems (37). Notably, in individuals with Down syndrome, MTRNR2L12 expression is significantly higher in adults who develop Alzheimer -like dementia compared to age-matched Down syndrome adults without dementia (17). The marked increase has led to the proposal that MTRNR2L12 may serve as a potential early blood-based or peripheral biomarker of Alzheimer-like dementia in Down syndrome patients (17). Although its precise role in dementia pathogenesis remains to be defined, the association suggests that MTRNR2L12 is implicated in neuronal stress adaptation.

MTRNR2L12 has been suggested to play a role in inflammatory disorders. In ulcerative colitis, higher expression of MTRNR2L12 and other MTRNR2L family members correlates with clinical remission, whereas decreased expression is associated with disease relapse (13). This pattern corroborates the experimental finding demonstrating an anti-apoptotic role of the predicted MTRNR2L12 peptide, HN12. In a Hirschsprung’s disease model, synthetic HN12 inhibited Bax translocation to mitochondria and reduced neuronal apoptosis (37), consistent with the established ability of humanin itself to suppress apoptosis by interfering with Bax activation (38). Because this evidence derives from synthetic HN12 and humanin peptides rather than an endogenously produced MTRNR2L12 product, it only demonstrates what the predicted peptide can do, but not that MTRNR2L12 is translated and protective *in vivo*. These findings suggest that MTRNR2L12 may contribute to tissue protection during inflammatory stress.

In AMD, scRNA-seq analysis revealed significant upregulation of MTRNR2L12 in macular vascular cell populations, particularly choroidal endothelial cells and smooth muscle cells, in eyes with intermediate AMD (**Table 2**) (30). Although the magnitude of MTRNR2L12 upregulation was smaller than that observed for MTRNR2L1, the coordinated increase across multiple vascular cell types suggests that MTRNR2L12 participates in mitochondrial and inflammatory stress response pathways activated in early AMD (30). Supporting this idea, MTRNR2L12 expression in RPE cells is higher in neovascular AMD compared to intermediate AMD, consistent with a role in cellular adaptation during disease progression (12). Together with the experimental evidence demonstrating anti-apoptotic and mitochondrial-preserving effects of MTRNR2L12, these findings point to MTRNR2L12 as a potential therapeutic target in retinal degenerative diseases, particularly those in which mitochondrial dysfunction and apoptosis drive pathology.

## Other MTRNR2L members

Beyond MTRNR2L1 and MTRNR2L12, additional paralogs have been investigated in various disease models. In ulcerative colitis, higher expression levels of MTRNR2L3, MTRNR2L6, MTRNR2L8, and MTRNR2L12 correlate with longer remission and reduced risk of disease relapse (13). Pathway enrichment analysis indicates that these paralogs participate in the negative regulation of apoptosis, suggesting protective roles in cell survival and stress resistance (13). In a single-cell RNA-seq study of peripheral blood mononuclear cells from pediatric patients with inflammatory bowel disease and eosinophilic gastrointestinal disorders, MTRNR2L8 was broadly upregulated across multiple immune cell types as a shared marker of cellular stress and active inflammation, with MTRNR2L1 additionally upregulated in the eosinophilic disorders (39). More recently, single-cell network analyses of pediatric high-grade gliomas identified MTRNR2L8 as a high-centrality node associated with mitochondrial stress adaptation and cellular plasticity in isocitrate dehydrogenase (IDH)-wildtype glioblastoma (31). These findings support a role for MTRNR2L8 as a biomarker of active inflammation and mitochondrial stress, and as a potential target gene for reducing chronic inflammation, an important pathogenic process in AMD and other retinal degenerative diseases. Interestingly, MTRNR2L6 expression in the RPE is increased in neovascular AMD compared to intermediate AMD, suggesting a possible role in AMD progression. In addition, several MTRNR2L genes, including MTRNR2L2, MTRNR2L3, MTRNR2L6, MTRNR2L8, MTRNR2L10, and MTRNR2L12, are more abundantly expressed in normal RPE compared to normal neuroretina, indicating that the MTRNR2L family may play an important role in maintaining RPE homeostasis and function.

Converging evidence from neurodegenerative disease further implicates the MTRNR2L family in stress-responsive neuroprotection. In a single-nucleus transcriptomic study of the prefrontal cortex across individuals with varying Alzheimer’s disease pathology, MTRNR2L8 and MTRNR2L12 were among the genes upregulated in disease, within a broader pattern in which genes induced at later pathological stages were shared across cell types and associated with a global stress response (14). A complementary picture emerges from a cerebral organoid model of tau-mutation (MAPT) tauopathy, in which MTRNR2L genes constituted the dominant component of the most enriched gene set among upregulated transcripts in mutant neurons, annotated to “negative regulation of execution phase of apoptosis,” an annotation that reflects gene-set enrichment rather than measured anti-apoptotic activity (14). Notably, the differential-expression data from that study reveal a dose-dependent pattern: across neuronal and astrocytic populations, MTRNR2L genes (including MTRNR2L1, MTRNR2L2, MTRNR2L3, MTRNR2L8, MTRNR2L10, MTRNR2L12, and MTRNR2L13) were consistently upregulated in heterozygous tau-mutant cells but downregulated in homozygous mutant cells carrying a more severe genotype, with MTRNR2L1 and MTRNR2L12 showing the largest reversals (15). This pattern suggests that MTRNR2L genes are activated as a protective, anti-apoptotic response under moderate cellular stress but fail to be sustained, falling below baseline, as the insult becomes more severe, although the organoid genotype-dosage design does not directly establish a temporal early-versus-late disease sequence. Together with the AMD, epilepsy, and inflammatory-disease findings discussed above, these observations position the MTRNR2L family as a coordinated cytoprotective stress-response system whose induction may mark a defensive, potentially reversible phase of neuronal and glial injury.

MTRNR2L3 has also emerged as a candidate serum biomarker in neuroinflammatory disease. Humanin-like 3, the peptide product attributed to MTRNR2L3, was measured by ELISA in the sera of patients with relapsing-remitting multiple sclerosis (MS), neuromyelitis optica spectrum disorder (NMOSD), and a clinically distinct MS subgroup presenting predominantly with optic nerve and spinal cord attacks (MS-SCON) (16). MS-SCON patients showed higher serum humanin-like 3 levels overall, with the difference reaching statistical significance versus NMOSD, and a 5.26 ng/ml cutoff discriminated MS-SCON from NMOSD with 66.7% sensitivity and 90% specificity (16). The authors proposed that elevated levels reflect a compensatory response to increased stress on neuronal survival in this subgroup, supporting the potential utility of MTRNR2L3 as a fluid-based biomarker of disease state. As this ELISA cannot distinguish MTRNR2L3 from other humanin-family peptides, the signal is best read as humanin-family immunoreactivity, and its attribution to MTRNR2L3 remains provisional.

## MTRNR2L genes and immune regulation

Beyond the trained-immunity and tumor associations noted above, two further lines of evidence connect MTRNR2L1 to immune and inflammatory processes of direct relevance to AMD. The first situates MTRNR2L1 within the immune compartment of the diseased choroid. In the single-cell analysis of MELAS retina and choroid discussed above, MTRNR2L1 was upregulated in three choroidal populations, namely fibroblasts, endothelial cells, and T cells, in the MELAS eye relative to control (27). Notably, the choroidal endothelial cells and T cells were near-homoplasmic for the wild-type mitochondrial allele, having selected against the pathogenic m.3243A>G variant, yet still upregulated MTRNR2L1 (27). This induction, occurring in cells that do not themselves carry a high mutational burden, suggests that MTRNR2L1 expression can be driven by the stressed tissue microenvironment rather than by cell-intrinsic mitochondrial dysfunction alone. The same cells co-upregulated TXNIP, a thioredoxin-interacting protein linked to redox stress (27).

The second connects these immune associations to a senescence-based mechanism directly relevant to AMD. Senescent RPE cells acquire a senescence-associated secretory phenotype (SASP) that releases IL-6, IL-8, and matrix metalloproteinases, driving the chronic, low-grade inflammation characteristic of AMD (40). NLRP3 inflammasome activation in RPE, an upstream event in this program, is promoted by TXNIP through reactive oxygen species (40), the same thioredoxin-interacting protein co-upregulated with MTRNR2L1 in the stressed MELAS choroid (27). Humanin itself counteracts this axis: in human RPE it reduces the senescence markers p16INK4a and ApoJ and roughly halves the fraction of senescence-associated β-galactosidase-positive cells under oxidative stress, through improved mitochondrial function and STAT3 signaling (41). Taken together, these observations support a testable hypothesis, that MTRNR2L induction in the AMD RPE and choroid represents a stress-adaptive attempt to restrain SASP output and the inflammation it drives, while leaving open whether MTRNR2L-encoded products themselves mediate this effect.

Across these areas, the available data are correlative, derive substantially from bulk or deconvolution-based analyses, and cannot at present distinguish whether MTRNR2L induction is a driver of, a response to, or a bystander of the immune states with which it co-occurs.

## Potential implications of MTRNR2L family in AMD pathogenesis

The evidence connecting the MTRNR2L family to AMD pathophysiology is thus far through gene expression, specifically from recent transcriptomic studies on human donor eyes (12, 30). Gene expression is the first step in ascribing function to a gene in disease, and differential expression measured directly in disease-affected tissue provides a more direct route to implicating a gene than genetic association alone, which identifies loci but rarely the causal gene or its mechanism (42–44). This is especially true for AMD, where the relevant transcriptional changes are concentrated in the macula and are best resolved in rigorously phenotyped donor eyes recovered at short post-mortem intervals (44, 45). In such tissue among MTRNR2L genes, MTRNR2L1 shows the strongest upregulation in the macula in both intermediate AMD and neovascular AMD compared to age-matched controls (12). Compared to the macula of intermediate AMD, a significant increase in MTRNR2L12, but not MTRNR2L1, was observed in the macula of neovascular AMD, suggesting differential roles for these genes in AMD development and progression (12). Although a cytoprotective effect of MTRNR2L12 has been demonstrated in cell culture (37), similar or distinct functions of MTRNR2L1 and other MTRNR2L genes remain unconfirmed. Given the significant sequence homology between MTRNR2L paralogs and humanin, along with the well-established ability of humanin to protect RPE cells from mitochondrial dysfunction, oxidative stress, and ER stress (46, 47), it is logical to hypothesize that MTRNR2L-encoded peptides may confer similar stress-resistance properties. Although further validation is needed, current evidence suggests that inhibition of intrinsic apoptosis by humanin and its analogs through stabilizing the mitochondrial membrane to prevent cytochrome C release and caspase 3 activation may contribute to their cytoprotective functions (48, 49).

Recent studies show that increased premature senescence of RPE cells, resulting in lipofuscin accumulation, drusen formation, and a pro-inflammatory senescence-associated secretory phenotype (SASP), is a key event in AMD pathogenesis (40). In human RPE cells, humanin counteracts cellular senescence by reducing expression of senescence markers such as p16INK4a and ApoJ transcripts (41) through improving autophagy and mitophagy, which facilitate clearance of damaged mitochondria and protein aggregates (50). It is possible that upregulation of MTRNR2L genes in AMD reflects a cellular defense response aimed at combating cellular senescence and dysfunction of the RPE. Future studies are needed to determine whether MTRNR2L genes possess similar effects on modulating autophagy and mitophagy and protecting against cellular senescence as humanin.

Beyond RPE, dysfunction of choroidal stromal cells, immune cells, and vascular cells also contributes critically to AMD pathogenesis. For example, abnormalities in choroidal melanocytes, the pigment-producing cells responsible for light absorption and regulation of free radical production, is linked to disturbed choroidal microenvironment, inflammation, and angiogenesis (51). Activation of immune cells, including mast cells, contributes to choroidal capillary loss and neovascularization in AMD (52). A recent scRNA-seq study found upregulation of MTRNR2L1 and MTRNR2L12 in macular choroidal vascular cells, Schwann’s cells, and melanocytes in AMD, consistent with a possible role for these genes in macular neovascularization driven by angiogenesis and inflammation (30). Additionally, increased MTRNR2L1 expression in choroidal endothelial cells, fibroblasts, and T cells in MELAS syndrome suggests that mitochondrial stress may induce MTRNR2L1 expression in these cells, linking mitochondrial dysfunction to choroidal inflammation, angiogenesis, and fibrosis in retinal degeneration and AMD.

The consistent upregulation of MTRNR2L1 and MTRNR2L12 in choroidal endothelial cells in intermediate AMD (**Table 2**) raises the possibility that these genes participate in maintaining the vascular microenvironment. Choroidal endothelial dysfunction is an early event in AMD, and factors that preserve endothelial survival and barrier integrity are therefore of particular interest. Humanin is expressed in the endothelial layer of human blood vessels and protects human aortic endothelial cells against oxidized-LDL-induced reactive oxygen species formation and apoptosis (53). In hypercholesterolemic ApoE-deficient mice, a humanin analog preserved endothelial function, reduced plaque apoptosis, and maintained aortic expression of endothelial nitric oxide synthase (eNOS), a central regulator of vascular tone, angiogenesis, and endothelial homeostasis (54). These effects are consistent with the PI3K/AKT and JAK/STAT3 signaling pathways that humanin activates in other cell types (20, 21). Given the close sequence homology between humanin and the MTRNR2L paralogs, it is reasonable to hypothesize that MTRNR2L-encoded peptides, if translated and functionally analogous, could similarly support choroidal endothelial survival and eNOS-dependent vascular homeostasis through PI3K/AKT signaling. This possibility remains untested and represents a specific, tractable direction for functional validation.

## Conclusions and perspective

Accumulating evidence supports an important role of the MTRNR2L family in the development and pathogenesis of human diseases, including AMD. Across all family members, MTRNR2L1 shows the strongest potential as a biomarker of mitochondrial stress and a candidate therapeutic gene, given its distinctive upregulation in macular RPE/choroid and neural retina in both intermediate and neovascular AMD (12, 30) and in choroidal vascular and immune cells in MELAS (27). The identification of MTRNR2L1 as a prognostic gene in tumor cells suggests a possible implication in stress-response and tumor cell survival. The involvement of MTRNR2L1 and MTRNR2L12 in neurological and inflammatory diseases further indicates that the MTRNR2L family may participate in regulating neuronal function and immune response (13–15, 17, 27, 28, 37).

Despite strong clinical evidence positioning MTRNR2L genes as candidates for cytoprotective pathways in retinal degeneration and AMD, several critical questions remain. First, do MTRNR2L genes encode proteins? Although the MTRNR2L family is traditionally considered pseudogenes, some pseudogenes are now known to encode functional peptides (55, 56). To date, no direct evidence demonstrates translation of any MTRNR2L genes. The broad disease associations suggest that these genes may function as non-coding RNAs, but this remains unproven. Second, what roles do MTRNR2L1 and MTRNR2L12 play in RPE and choroidal cells during AMD? Gain-of-function and loss-of-function studies are needed to determine how they influence cell survival and delineate which signaling pathways they engage. Third, can MTRNR2L genes serve as biomarkers for mitochondrial stress or disease progression in AMD and beyond? MTRNR2L12 has been proposed as a blood marker for Alzheimer-like dementia in Down syndrome (17). Testing whether MTRNR2L transcripts or peptides are detectable in serum or ocular fluids across AMD stages could provide valuable diagnostic tools. Recent evidence that serum humanin-like 3 (associated with MTRNR2L3) discriminates an optic-nerve and spinal-cord-predominant MS subgroup from NMOSD lends preliminary support to the serum-biomarker concept (16). Finally, can MTRNR2L genes serve as therapeutic targets? Evidence from humanin and its analogs, such as Humanin-G, demonstrates strong cytoprotective effects in mitochondrial stabilization, reduction of oxidative and ER stress, activation of pro-survival signaling, and inhibition of apoptosis in RPE cells (46–49, 57). The potential involvement of MTRNR2L genes in angiogenesis and inflammation also warrants further exploration.

Underlying all of these questions is a methodological one. Because the current evidence rests almost entirely on transcript-level measurements, and because the MTRNR2L family is closely tied to mitochondrial metabolism and cellular bioenergetics, transcriptomic data alone cannot establish whether MTRNR2L induction produces meaningful downstream metabolic consequences. Integrated multi-omics approaches will be important to close this gap. In particular, pairing transcriptomics with metabolomics in stressed RPE or choroidal endothelial cells would allow the transcriptional induction of MTRNR2L genes to be mapped onto specific shifts in metabolite pools, including amino acid metabolism and other pathways linked to mitochondrial function, and would help distinguish an adaptive metabolic role from an incidental transcriptional response.

In summary, the MTRNR2L family represents an exciting opportunity to uncover new regulatory mechanisms of mitochondrial health and cell survival in AMD. Addressing the remaining questions will be essential for understanding their biological functions, regulatory mechanisms, and disease relevance. If future studies confirm their therapeutic promise, MTRNR2L-directed approaches, such as peptide analogs, gene augmentation, or pathway-level modulation, could serve as biomarkers of disease severity or as therapeutic strategies that complement existing AMD treatments, potentially delaying disease progression by preserving RPE and choroidal cell function and homeostasis.
